# Accuracy of Tumor Perfusion Assessment in Rat C6 Gliomas Model with USPIO

**DOI:** 10.1515/med-2019-0091

**Published:** 2019-11-07

**Authors:** Xiang-Rong Yu, Bo-Ling Cao, Wei Li, Ye Tian, Zhong-Li Du

**Affiliations:** 1 Zhuhai Hospital of Jinan University, Zhuhai People’s Hospital, Zhuhai, 519000, China; 2 Department of Radiology, Zhuhai Hospital of Jinan University, Zhuhai People’s Hospital, Zhuhai, China

**Keywords:** Perfusion imaging, USPIO, Gd-DTPA, Rat, Glioma

## Abstract

Detailed characterization of the permeability and vascular volume of brain tumor vasculature can provide essential insights into tumor physiology. In this study, we evaluated the consistency of measurements in tumor blood volume and examined the feasibility of using ultrasmall superparamagnetic iron oxide (USPIO) versus gadolinium-diethylene triamine pentaacetic acid (Gd-DTPA) as contrast agents for MR perfusion imaging of brain gliomas in C6 Rats. Eighteen rats were intracerebrally implanted with C6 glioma cells, randomly divided into two groups and examined by 3.0T perfusion MR imaging with Gd-DTPA and USPIO. Tumor relative cerebral blood volume (rCBV) and relative maximum signal reduction ratio (rSRRmax) were created based on analysis of MR perfusion images. The mean values for rCBV were 2.09 and 1.57 in the USPIO and the Gd-DTPA groups, respectively, and rSRRmax values were 1.92 and 1.02 in the USPIO and the Gd-DTPA groups, respectively, showing signifi cant differences in both rCBV and rSRRmax between the USPIO and the Gd-DTPA groups (P < 0.05). The results showed that early vascular leakage occurred with gadolinium rather than USPIO in perfusion assessment, revealing that USPIO was useful in perfusion MR imaging for the assessment of tumor vasculature.

## 1 Introduction

Angiogenesis is an essential feature of brain tumors and has served as a target for multiple novel therapies. Currently, conventional T2-weighted and contrast enhanced T1-weighted magnetic resonance (MR) imaging sequences are reliable for evaluating tumor angiogenesis and monitoring antiangiogenic therapies. An alternative technique is to measure cerebral blood volume (CBV) with dynamic susceptibility-weighted contrast enhanced perfusion MR imaging. Previous studies show that MR perfusion imaging is a valuable adjunct to conventional imaging, and perfusion imaging correlates with tumor progression [1, 2, 3, 4]. The estimation of relative CBV (rCBV) with this method relies on the assumption of an intact blood brain-barrier (BBB) which might be affected by glioma. Disruption of the BBB can increase the permeability of vasculature, leading to leakage of low molecular weight contrast gadolinium diethylene triamine pentaacetic acid (Gd-DTPA) from the vascular system into the interstitial space. The leakage of Gd -DTPA can result in changes in interstitial signal intensity, which confounds changes in intravascular susceptibility-induced signal intensity during passage of the contrast agent through the vasculature [5] and leads to the underestimation of CBV [6, 7], especially in lesions with highly permeable vasculature [8, 9, 10].

To improve the performance of perfusion MR imaging in diagnosis, in this work a blood pool contrast agent, ultrasmall superparamagnetic iron oxide particles (USPIO), was used to characterize the tu mor vasculature and evaluate the consistency of tumor blood volume measurements in C6 rat glioma models using perfusion MR imaging in comparison with Gd-DTPA [11, 12, 13]. The USPIO contrast agent was examined in the intravascularature for a prolonged period of time and transverse water proton MR relaxation rate was tested, with T2 relaxivity of USPIO being up to 20 times of that of Gd-DTPA [13]. USPIO may be a reliable marker for blood volume estimation in le sions with BBB disruption.

## 2 Materials and methods

### 2.1 Animal Procedures

Adult male SD rats (275±25 g, n = 18) were purchased from Shanghai SLAC Lab Animal Ltd. (Shanghai, China) and maintained under standard conditions. The animal experiments were conducted under the regulation of the ethics committee of Jinan University and we have complied strictly with all the rules associated with animal study. C6 cells were cultured in Dulbecco’s modified Eagle’s medium (DMEM; Gibco, Gaithersburg, MD) with 5% fetal calf serum, 1% penicillin-streptomycin and 1% glutamine in a humidified incubator at 37°C with 5% CO_2_. For implantation, nearly confluent C6 glioma cells were harvested and resuspended in DMEM at the density of 1×10^6^ cells in 10μL of culture medium. Cells were inoculated into the right striatum as follows. Rats were anesthetized with 10% Chloral Hydrate (3.5 mL/ kg) via intraperitoneal injection (i.p.), and immobilized in a stereotactic head set with ear bars and a teeth bar. The skull was exposed by a 2 cm midline incision, and a burr hole was created on the right side 1 mm anterior and 3 mm lateral to the bregma. 10 μL of DMEM containing 1×10^6^ C6 cells was injected from the skull surface into the frontal lobe at a depth of 5 mm over a period of 5 minutes. The needle was kept in place for 5 minutes after injection to prevent backflow prior to removal. After surgery, the burr hole was filled with bone wax and the scalp was sutured.

### 2.2 MRI Acquisition

After tumor inoculation all rats (n = 18) were randomly divided into two groups (9 rats each) and characterized with MRI at day 12. The effective sample size in each group was calculated based on the resource equation method proposed by Festing et al [14, 15] and followed the guidelines proposed by Charan and Kantharia [16]. Rats were anesthetized with Chloral Hydrate (3.5 mL/kg) via intraperitoneal injection. After 5 minutes the rats were catheterized in the tail vein and placed in a clinical 3.0-T MR system (Signa HDX, GE Medical System, LLC, USA) with a 3-inch-diameter 4-element phased array coil. During imaging, rats were wrapped by a warm water blanket under saturated oxygen and heart rate was monitored simultaneously. Before USPIO injection, dextran coated 4-6 nm iron oxide (20-30 nm in diameter, Sinerem, Guerbet Laboratories, Aulnay-Sous-Bois, France), was diluted in 0.9% NaCl. The two groups were injected with USPIO and Gd-DTPA (Magnevist Schering, Berlin, Germany) at 0.2 mM/kg via tail vein, respectively, which was conducted within 1s of the fourth imaging occurring, then followed immediately by a 0.3 mL of saline flush at a rate of 1 mL/ min. Coronal imaging sequences included: spin-echo T1 weighted imaging and fast spin-echo T2 weighted imaging. Dynamic susceptibility-weighted contrast enhanced perfusion MR images were acquired by using a gradient-echo pulse sequence. After rapid bolus administration of contrast agent was performed via the tail vein, we performed three consecutive DSC perfusion MR to acquire 45 volumes within ~3 minutes. Then conventional contrast enhanced T1-weighted imaging was conducted immediately in the Gd-DTPA group, and fast spin-echo T2 weighted imaging was performed 12 hours later in the USPIO group.

### 2.3 Histological analysis

After MR imaging, rats were sacrificed with CO_2_, followed by heart perfusion with saline and 4% paraformaldehyde. Brains with glioma legions were cut and the glioma volume was calculated by the formula: V= 1/2×a×b^2^, where a and b represented the maximum and minimum diameters of glioma, respectively. Then glioma-bearing brains were embedded in optimal cutting temperature compound and frozen while fresh. Serial 5-μm thick sections of glioma-bearing brains were obtained and stained with hematoxylin and eosin (HE) and Prussian blue, respectively, according to standard clinical pathology protocols. Immunohistochemical assays of C6 glioma preparations were performed with primary rabbit polyclonal antibody to glial fibrillary acidic protein (GFAP; dilution, 1:500; Abcam USA). Tumoral vascular cells were stained with primary rabbit polyclone CD 31 (dilution, 1:100; Abcam USA) at 4°C overnight, and further stained with Alexa Fluor 647-conjugated goat anti-rabbit secondary antibody (dilution, 1:100; Jackson USA) for 1 h at room temperature. Cell nuclei were stained by Hoechst 33342 (1 mg/ ml, Nantong China) at room temperature for 10 min. The number of tumoral vessels was counted in five high power field areas. Transmission electron microscopy (Philip CM-120, Eindhoven Netherlands) was used to observe the ultrastructural changes of the tight junctions and pinocytotic vesicles in glioma microvascular endothelial cells.

### 2.4 Image Processing

All data were acquired and processed by a SPARC (Sun Micro-systems) workstation. The intensity-time curve and raw data were obtained based on signal-time curve from perfusion MR imaging. Maximum signal reduction ratio (SRRmax) was calculated as below: SRRmax=(SIpre-SImin) /SIpre×100%, where SIpre is the mean signal intensity prior to injection of contrast agent and SImin is the minimal signal intensity during the process of post-contrast perfusion. Changes in the SRRmax were calculated in the different regions of interests (ROIs) for each group. A fully automated deconvolution analysis was performed to create parametric images of CBV. To quantify changes of CBV and SRRmax in tumor and normal tissues, color-coded CBV maps were created on a voxel-wise basis and projected onto the T2- weighted images, which provided better visualization of tumor boundaries. To minimize potential measurement biases, the images were randomly provided and necrotic areas were excluded. Two radiologists drew a ROI by consensus with the highest CBV areas on the perfusion maps within the three portions of the tumor lesion, and the averaged ROI values were obtained as the mean value of the tumor. Furthermore, the radiologists evaluated the contralateral normal tissue in a normal-appearing color-coded CBV map with the same size ROI (2 mm minimum diameter; circular ROI at least 4 mm^2^). Relative ratios of CBV and SRRmax in the tumor against those in the contralateral brain tissue were calculated.

### 2.5 Statistical Analysis

All continuous variables were expressed as means±stand-ard deviations. The size differences of the glioma between the USPIO group and the Gd-DTPA group were examined using the independent-samples t-test. The differences in CBV and SSRmax in the tumor lesion and normal brain tissue were examined by paired-samples t-test. The differences in relative ratios of CBV and SRRmax between the USPIO group and the Gd-DTPA group were examined using the independent-samples t-test. All statistical analyses were performed with SPSS software (SPSS for

Windows, version 16.0; Chicago, IL). A *P* value less than 0.05 was considered as statistically significant.

## 3 Results

### 3.1 Imaging manifestation

**Figure 1:**
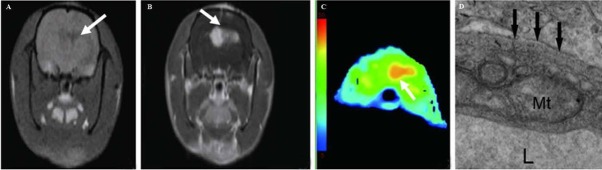
C6 glioma assessed with Gd-DTPA. A: T1-weighted image showed a hypointense tumor without distinct border (arrow). B: After administration of Gd-DTPA, A heterogeneous enhancement tumor with necrosis was noted (arrow). C: The mean of highest CBV areas(arrow) was 21.35 with administration of Gd-DTPA in the tumor. D: TEM image showed significant increase of the pinocytotic vesicles and opening tight junctions (arrow).

**Figure 2:**
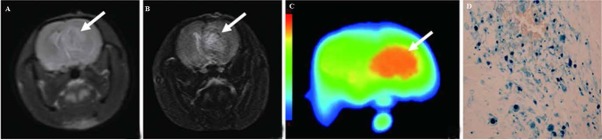
C6 glioma assessed with USPIO. A: T2-weighted image showed a hyperintense tumor without distinct border (arrow). B: After administration of USPIO, the T2 signal decreased in the periphery of the tumor. Tumor microvessels were more readily identified as hypointense serpiginous structures within the tumor (arrow). C: The highest CBV areas were prominent on color-coded CBV maps. D: Sections were stained with Prussian blue after administration of USPIO, showing that iron particles located in capillaries in the border zone of the necrotic lesion.

At 12 days after tumor inoculation, the glioma volumes were calculated to be 58.52 ± 6.34 mm^3^ and 57.45 ± 6.62 mm^3^ for the USPIO and the Gd-DTPA groups, respectively, indicating no significant differences in tumor sizes between groups. The tumors showed as hypointense on T1 weighted images (Fig 1A) while showing as hyperintense on T2 weighted images (Fig 2A). Necrosis was seen on MR images in all the rats. After administration of Gd-DTPA and USPIO, tumors were easily identified. Every tumor model displayed a distinctive pattern of vascular morphology and enhancement after administration of contrast agents. With Gd-DTPA administration, all tumors were hyperintense on T1 weighted images, indicating extravasation of Gd-DTPA (Fig 1B). With administration of USPIO, all tumors presented as hypointense on T2 weighted images. The negative enhancing effect presented first at the border of the tumors, and then infiltrated toward the center gradually. Tumor microvessels were readily identified as hypointense serpiginous structures within the tumor (Fig 2B).

### 3.2 Perfusion MR imaging findings and histomorphometry

CBV and SSRmax values of tumor and contralateral tissue from the USPIO and Gd-DTPA groups are presented in Table 1. Average CBV values obtained in tumors were all larger than those obtained in contralateral tissue (*P* < 0.01) (Figs 1C, 2C). Differences of SSRmax between tumor and contralateral tissues were significant (*P* < 0.01).

The relative ratios of CBV(rCBV) in the USPIO group varied from 1.78 to 2.58 with a mean of 2.09±0.23 (SD), whereas in the Gd-DTPA group ratios varied from 1.22 to 1.86 with a mean of 1.57±0.19. The relative ratios of SSRmax (rSSRmax) in the USPIO group varied from 1.33 to 2.32 with a mean of 1.92±0.37, while in the Gd-DTPA group ratios varied from 0.81 to 1.42 with a mean of 1.02±0.27. A significant difference existed in rCBV and rSSRmax between the USPIO and the Gd-DTPA groups based on the two-tailed independent-samples T test (*P* < 0.05).

**Figure 3:**
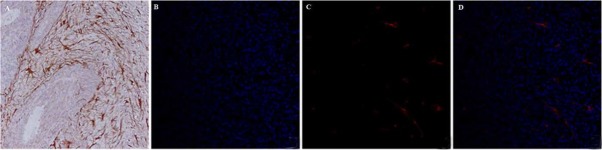
C6 glioma assessed with histological analysis. A: Numerous cells expressed GFAP. B: Blue fluorescence showed the cell nuclei. C: Red fluorescence showed vascular. D: Merged imaging depicted the colocalization of nuclei and vascular in tumor area. In addition, 35.5±6.2% vascular (red) in tumor lesion, which was significantly higher than that in normal tissue.

All tumors exhibited the invasive growth of gliomas and significant increases of the pinocytotic vesicles and opening tight junctions (Fig 1D). Prussian blue staining was carried out to further verify the accumulation of the USPIO in turmor legions. As shown in Fig 2C, blue spots of USPIO were observed in tumor region. Immunohisto-chemistry revealed that numerous cells expressed GFAP presented as brown granules, suggesting the presence of glioma (Fig 3A). Vascularature was immunofluorescence-stained with CD 31 antibody, showing 45.5±6.2%

**Table 1:** Comparison of perfusion metrics obtained for rat models with different imaging methods (n=9)

Metrics	Method	Tumor region	Normal tissue	Relative ratio
CBV	USPIO	33.53±2.85	17.28±1.51	2.09±0.23
	Gd-DTPA	23.35±2.14	17.47±1.64	1.57±0.19
SRRmax	USPIO	28.99±3.23	15.07±2. 03	1.92±0.37
	Gd-DTPA	17.90±2.59	15.30±1.79	1.02±0.27

neovascularature in tumor lesions, which was significantly higher than that in normal tissue (Figs 3B-D).

## 4 Discussion

Tumor vascularity is closely associated and might be mutually promoted in glioma growth. In our study, the immunofluorescence staining of tumor vascularature showed 45.5±6.2% neovascularature in tumor lesions, which was significantly higher than that in normal tissue. Noninvasive evaluation of the permeability and vascular volume of tumor vasculature can provide essential insights into tumor physiology, which is the prerequisite to investigate and evaluate tumor responses to antiangiogenic therapies. Evidence showed that MR perfusion imaging was a sensitive marker for the tumor vascularity [17]. However, normal brain tissues are protected by a functional blood brain barrier (BBB) but glioma regions are characterized by an impaired BBB. Blood volume estimation would be more reliable in lesions with disrupted BBB when a blood pool contrast agent is used to minimize extravasation artifacts. Traditional MRI techniques rely on gadolinium (Gd) to demonstrate defects in the BBB caused by tumors because Gd can rapidly cross disrupted BBB into extracellular space and be cleared from the blood circulation due to its small molecular size [10,18]. Unlike Gd, USPIO initially remains in the intravascular space after administration due to its large size, potentially allowing more accurate perfusion imaging. Here, we adopted dynamic susceptibility-weighted contrast enhanced perfusion MR imaging, one of the most common tumor vasculature assessment techniques, to evaluate the efficacy of Gd-DTPA versus USPIO in C6 rat glioma models.

Measurements of rCBV closely correlate with angiographic and histologic markers of tumor vascularity, in which a threshold value greater than 1.75 in gliomas has been widely used in previous studies and clinical practice [4,10,19]. Our results showed that five rats with diffuse infiltrative gliomas showed low rCBV (≤ 1.75) with Gd-DTPA while all nine rats showed high rCBV (>1.75) with USPIO, showing significant difference of rCBVs between Gd-DTPA and USPIO. Gahramanov et al [10] reported similar results by performing three consecutive perfusion MR imaging acquisitions in U87MG rat models, which were performed without preload, with single-dose preload, and with double-dose preload. Perfusion MR imaging with gadodiamide and without preload showed low rCBV (≤ 1.75) in 9 of 13 tumors and estimated rCBV increases progressively with both single- and double-dose preloads (*P* <.001). Conversely, rCBVs obtained with ferumoxytol were high (>1.75) and remained constant in all three acquisitions. This finding is consistent with previous clinical imaging results by Hu et al [20] who performed six consecutive perfusion MR image acquisitions with gadolinium-based contrast agent and correlated rCBVs with histopathologic findings in patients with high-grade glioma after chemoradiotherapy. Their results showed rCBV is underestimated in biopsy verified tumors and is dependent on preload dose of contrast agent. All of the results indicated that tumor rCBV based on gadolinium-based contrast agent can be underestimated without preload and is dose-dependent with preload correction. Conversely, USPIO provides consistent assessment of tumor rCBV, which provides an objective comparison of vascular volumes among different tumors and allows performance of longitudinal studies to assess various factors, such as treatment efficacy.

In addition to vascular volume measurements, we have derived an image metric that reflects the permeability and extracellular volume of brain tumor vasculature modeled from perfusion MR imaging, showing that this metric (SRRmax) can be done by visually evaluating the changes in signal intensity. Specifically, the USPIO group with a higher relative SSRmax is more likely to have a lower vascular leakage than the Gd-DTPA group in our study. In the current study, significant increases of the pinocytotic vesicles and opening tight junctions in glioma microvascular endothelial cells is observed in the TEM results. Previous studies showed an increase of vascular permeability, accompanied with the increase of the pinocytotic vesicles and opening tight junctions [21, 22, 23]. The healthy capillary with an intact BBB is impermeable to the contrast agent and remains intravascularly, due to which the signal intensity should return to around baseline value. A diseased capillary with a disrupted BBB might be permeable to the contrast agent, leading to the accumulation of contrast agent outside the vessel. The changes of SRRmax were recognized as an approximation of vasculature permeability to the contrast agent [19]. The leakage of contrast agent out of the vessel into the extravascular space of the tissue can cause the progressive decrease of SRRmax. In addition, leakage of Gd-DTPA from the tumor is not restricted to the tumor interstitium but can extend into the surrounding normal tissue, which probably precludes an accurate estimation of the tumor edges. Thus, owing to the large size of the iron nanoparticles and a long half-life, the inability of USPIO to cross the disrupted BBB in the short term provides an attractive alternative to Gd-DTPA for blood volume measurements [18].

There were some limitations in our study that must be considered. Perfusion MR imaging provides a measurement of tumor blood volume as a ratio compared with normal-appearing parenchyma rather than an absolute measurement of tumor vasculature as a percentage of tumor volume. There is, however, evidence that measurements of rCBV correlate closely with angiographic and histologic markers of tumor vascularity [17,21]. In our study, we only compared USPIO with Gd-DTPA in terms of vasculature and permeability of tumors. In our future study, evaluation of the efficacy of antiangiogenic therapy with USPIO versus Gd-DTPA by perfusion MR imaging will be evaluated. Furthermore, it will be instructive to see if rCBV and rSRRmax can give predictable responses to the therapies, which needs clarifying with future study. Finally, mathematical correction of contrast agent extravasation is not applied during postprocessing. It is well known that rCBV is underestimated with Gd-DTPA, and a number of mathematical algorithms have been proposed to minimize artifacts from contrast agent extravasation. These methods, however, require sophisticated postprocessing and suffer from a lack of consistency, according to Paulson et al [5] who compared five different mathematical correction algorithms in the same patient and demonstrated dependence of variability in tumor rCBV on the choice of methods of analysis.

## 5 Conclusion

We have characterized in detail the tumor vasculature by imaging vascular leakage and vascular volume. Our results show that USPIO has advantages in the assessment of tumor perfusion due to its low leakage rate, demonstrating that USPIO is a highly attractive alternative to Gd-DTPA in MR perfusion imaging.
